# Temperature-dependent release of ATP from human erythrocytes: mechanism for the control of local tissue perfusion

**DOI:** 10.1113/expphysiol.2011.064238

**Published:** 2012-01-13

**Authors:** Kameljit K Kalsi, José González-Alonso

**Affiliations:** Centre for Sports Medicine and Human Performance, Brunel UniversityUxbridge, Middlesex, UK

## Abstract

Human limb muscle and skin blood flow increases significantly with elevations in temperature, possibly through physiological processes that involve temperature-sensitive regulatory mechanisms. Here we tested the hypothesis that the release of the vasodilator ATP from human erythrocytes is sensitive to physiological increases in temperature both *in vitro* and *in vivo*, and examined potential channel/transporters involved. To investigate the source of ATP release, whole blood, red blood cells (RBCs), plasma and serum were heated *in vitro* to 33, 36, 39 and 42°C. *In vitro* heating augmented plasma or ‘bathing solution’ ATP in whole blood and RBC samples, but not in either isolated plasma or serum samples. Heat-induced ATP release was blocked by niflumic acid and glibenclamide, but was not affected by inhibitors of nucleoside transport or anion exchange. Heating blood to 42°C enhanced (*P* < 0.05) membrane protein abundance of cystic fibrosis transmembrane conductance regulator (CFTR) in RBCs. In a parallel *in vivo* study in humans exposed to whole-body heating at rest and during exercise, increases in muscle temperature from 35 to 40°C correlated strongly with elevations in arterial plasma ATP (*r*^2^ = 0.91; *P* = 0.0001), but not with femoral venous plasma ATP (*r*^2^ = 0.61; *P* = 0.14). *In vitro*, however, the increase in ATP release from RBCs was similar in arterial and venous samples heated to 39°C. Our findings demonstrate that erythrocyte ATP release is sensitive to physiological increases in temperature, possibly via activation of CFTR-like channels, and suggest that temperature-dependent release of ATP from erythrocytes might be an important mechanism regulating human limb muscle and skin perfusion in conditions that alter blood and tissue temperature.

Internal body temperature is a highly regulated physiological process in humans and other homeothermic animals. However, large regional differences in temperature still exist between internal organs and limb tissues, with the latter being 2–5°C lower in humans and larger animals in normal resting conditions (Bazett & McGlone, 1927; He *et al.* 2002). These temperature differences are also highlighted during exercise, when the temperature of the blood and muscle of the exercising limbs can increase from 33–35 to 40–41°C while in non-exercising limbs it remains essentially unchanged (Saltin *et al.* 1972; González-Alonso *et al.* 1999*a*,*b*). Hence, RBCs can be exposed to drastic changes in temperature as they travel through tissues with different metabolic heat production and haemodynamic demands. Although recent evidence in heat-stressed humans supports a close association between the elevations in limb tissue perfusion and the increases in muscle temperature and arterial plasma adenosine 5′-triphosphate (ATP; Pearson *et al.* 2011), the ATP source and temperature-sensitive mechanisms involved remain unknown.

The erythrocytes, the major oxygen carriers in the blood, have been hypothesized to play a crucial role in the control of local tissue blood flow. According to the hypothesis proposed by Ellsworth *et al.* (1995), when the erythrocytes encounter an area where metabolic demands are augmented a signalling mechanism coupled to the offloading of oxygen is triggered, resulting in the release of ATP from the erythrocytes into the vascular lumen. The ATP acts upon the endothelial P_2y_ receptors, triggering the release of nitric oxide, prostaglandins and/or endothelium-derived hyperpolarizing factor, which in turn act upon the surrounding smooth muscle cells to cause vasodilatation (Ellsworth *et al.* 1995; Sprague *et al.* 1996; Mortensen *et al.* 2009*a*,*b*). The release of ATP from erythrocytes can occur not only in response to a reduction in 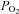, but also in response to blood cell deformation (Sprague *et al.* 1998; Fischer *et al.* 2003). The endothelium could be another source of ATP; however, catabolic ectonucleotidases (Zimmermann, 2006*b*) present on the surface of cellular membranes (Gordon, 1986; Zimmermann, 2006*a*) immediately convert intravascular ATP to adenosine, thereby evoking P_1_ receptor-mediated vasodilatation (Deussen *et al.* 1986). It has long been known that an increase in temperature reduces the affinity of haemoglobin for oxygen (Barcroft & King, 1909; Duc & Engel, 1969). This suggests that temperature has the potential to modulate the release of ATP from erythrocyte directly or indirectly; however, no study to date has systematically investigated whether temperature *per se* is a major stimulus for the release of ATP from erythrocytes.

The mechanisms of ATP release from erythrocytes are thought to involve membrane-bound ion channels, gap junction proteins, such as pannexin 1, and/or members of the ATP-binding cassette proteins (ABC proteins), such as the cystic fibrosis transmembrane conductance regulator (CFTR; Bergfeld & Forrester, 1992; Abraham *et al.* 1993; Locovei *et al.* 2006). The impact of temperature on these channels/transporters is not known. The membrane-bound ion channel known as band 3 (also known as the anion exchanger AE1) was the first channel proposed to regulate the release of ATP from erythrocytes with exposure to hypoxia (Bergfeld & Forrester, 1992). More recently, the gap junction protein pannexin 1, which is also abundantly expressed in erythrocytes, has been postulated to form ATP-permeable channels in the plasma membrane, and responds to low oxygen tension through its action on the signal transduction pathway leading to ATP release (Locovei *et al.* 2006; Sridharan *et al.* 2010). Lastly, the CFTR channels in erythrocytes and other cells have been shown to be activated by external physiological stimuli, such as cell deformation, cell swelling and changes in pH (Sprague *et al.* 1998; Gourine *et al.* 2010; Tu *et al.* 2010). Whether the aforementioned channels/transporters are involved in the release of ATP from erythrocytes when temperature is increased has never been examined.

The main purpose of this study, therefore, was to investigate the source and the temperature-sensitive mechanism of ATP release in human blood. To accomplish this overall aim, the following investigations were carried out: (i) whole blood and its separate constituents were heated *in vitro* to establish the primary source of ATP; (ii) specific and non-specific channel inhibitors were used to block *in vitro* ATP release from human erythrocytes to understand the mechanism of heat-induced ATP release; (iii) blood samples from healthy volunteers exposed to heat stress in resting and exercising conditions were assessed to examine whether ATP release was comparable to the response observed in our *in vitro* experiments; and (iv) arterial and venous blood was heated *in vitro* to assess whether the oxygenation status of the blood affects the amount of ATP release. We hypothesize that the release of ATP from human erythrocytes is sensitive to physiological increases in temperature *per se*, both *in vitro* and *in vivo*, and that channels localized in the erythrocyte membrane are involved in this process.

## Methods

This study, consisting of five *in vitro* protocols and one *in vivo* protocol (i.e. protocols 1–6) conformed to the code of Ethics of the World Medical Association (Declaration of Helsinki) and was conducted after receiving ethical approval from the Brunel University Research Ethics Committee. Informed written and verbal consent was obtained from all of the participants before commencing with any part of this study. Subjects were asked to refrain from exercise and ingestion of caffeine on the day of blood withdrawal.

Blood samples for the *in vitro* heating protocols 1–4 were obtained by venepuncture of an antecubital vein in 27 healthy men ranging in age from 21 to 46 years (mean ± SD age 28 ± 7 years) and were tested on the day of collection (within 30–50 min of blood collection). Blood was always collected in a syringe and immediately aliquoted into K3-EDTA or serum separation tubes. For protocol 5, 10 healthy recreationally active men (mean ± SD age 21 ± 2 years) participated in a graded whole-body heating protocol, which has been described in detail previously (Pearson *et al.* 2011). Only during this protocol, arterial and venous blood samples were assayed immediately after collection. In protocol 6, arterial and venous blood was collected from four subjects (mean ± SD age 31 ± 6 years) in a syringe and immediately aliquoted into K3-EDTA. The red blood cells (RBCs) were then heated *in vitro*.

### Protocol 1: effect of heating on ATP release from blood constituents *in vitro*

The main aim of this protocol was to determine the source of ATP by heating whole blood and the separate constituents of blood. Blood was collected from 10 individuals for this part of the study. Blood collected in EDTA tubes was separated into eppendorf tubes and kept as whole blood or centrifuged at 15,493*g* for 30 s at room temperature. Plasma was removed and transferred into new tubes; the buffy coat was removed and discarded, while the RBC fraction was also kept for experimentation. The volume was restored by adding physiological saline after plasma and buffy coat removal from the RBC fraction. Tubes used for serum separation were coated with silicon and micronized silica particles to accelerate clotting (SST II advance serum separation tubes; Becton & Dickinson, Oxford, UK). Blood was allowed to clot for 30 min at room temperature, and serum was collected after centrifugation for 10 min at 1000*g*. Aliquots (0.5 ml) of whole blood, RBCs, plasma and serum were heated for 20 min (Baar, 1967) in stirred water baths set at 33 (control), 36, 39 and 42°C. Prior to the heating protocol, initial levels of ATP and free haemoglobin were measured. To further determine the effects of heating on whole blood haematological parameters, RBC count, total haemoglobin, haematocrit, mean cell volume, mean cell haemoglobin and mean corpuscular haemoglobin concentration were measured using an automatic haematology analyser (Sysmex KX-21N; Sysmex UK Ltd, Milton Keynes, UK) after repeated blood samples from one individual were incubated for 20 min at 33, 36, 39 and 42°C.

### Protocol 2: time course for *in vitro* heat-dependent erythrocyte ATP release

The aim of this protocol was to ascertain the time course of the increase in ATP release from human erythrocytes with heating over 20 min. Tubes with RBCs were initially placed in a water bath set at 33°C, where the temperature was continuously monitored by a thermocouple inserted into a tube containing RBCs. Once the temperature was stable at 33°C for ∼5 min, a set of tubes containing RBCs were moved to a water bath maintained at 42°C. Another tube containing RBCs with a second thermocouple was used to indicate the time needed to reach 42°C starting from 33°C. Samples were removed from the water baths set at 33 and 42°C after 1, 3, 5, 10, 15 and 20 min. At each time point, the temperature was noted and samples were rapidly prepared for ATP analysis. Data were collected from six to 10 healthy individuals.

A further set of RBC samples (*n* = 6) were heated to 42°C for 20 min and then moved to a water bath set at 33°C. Samples were removed at regular intervals for ATP analysis. Release of ATP was compared with control samples kept at 33°C. In these sets of experiments, the number of RBCs was counted using a haemocytometer for samples before and after 20 min of heating.

### Protocol 3: effect of inhibitors of ATP channels and transporters on heat-dependent erythrocyte ATP release

In order to distinguish the potential channel or transporter involved in the heat-dependent ATP release from erythrocytes, various inhibitors were used to block the release during *in vitro* heating. Isolated RBCs were incubated for 20 min in water baths set at 33 (control) and 39°C with various inhibitors of band 3 and band 4.5. The inhibitors used to block band 3 transport included niflumic acid, which was dissolved in physiological (0.9%) saline, and DIDS, dissolved in 2% DMSO. The inhibitor used to block band 4.5 nucleoside translocation was nitrobenzylthioinosine (NBTI) dissolved in DMSO. Stock solutions of inhibitors were prepared so that the final concentration of DMSO was less than 1% and did not induce haemolysis.

Red blood cells isolated from arterial and venous blood samples, collected during protocol 6, were incubated for 20 min in water baths set at 33 (control) and 39°C with 400 μmol l^−1^ niflumic acid or 400 μmol l^−1^ glibenclamide (an ATP-sensitive potassium channel blocker which also blocks CFTR; Sheppard & Robinson, 1997). Glibenclamide was prepared as a 0.01 mol l^−1^ stock solution in 0.1 n NaOH and 50 mg ml^−1^ dextrose solution and heated at 52°C to dissolve (Sprague *et al.* 1998). The effect of vehicle alone was also tested. All chemicals were purchased from Sigma-Aldrich (Gillingham, UK).

### Protocol 4: effect of *in vitro* heating on CFTR abundance in erythrocyte ghost membrane isolation

The purpose of this protocol was to investigate whether CFTR protein in the membranes of erythrocytes was involved in the heat-dependent release of ATP. Human red blood cell ghost membranes were prepared from RBCs according to the method of Sterling *et al.* (2004) after they were exposed to 33 and 42°C for 20 min. The purified RBC membranes were dissolved using 50 mmol l^−1^ Tris–HCl (pH 7.5), 150 mmol l^−1^ NaCl, 1% Triton X-100 and 1% Nonidet P-40 plus protease inhibitor cocktail. For the enzyme-linked immunosorbent assay (ELISA), a microtitre 96-well plate was coated with the dissolved membrane extracts in carbonate buffer (15 mmol l^−1^ Na_2_CO_3_ and 35 mmol l^−1^ NaHCO_3_, pH 9.6) and incubated overnight at 4°C. Blocking buffer of 2% bovine serum albumin in PBS was added and incubated for 1 h at 37°C. After washing with PBS, wells were incubated with rabbit polyclonal antibodies raised against the amino terminus of human CFTR diluted 1:2000 in PBS (New England Biolabs, Hitchin, UK) for 1 h at 37°C and 2 h at 4°C. After washing with 0.02% Tween 20 in PBS, wells were treated with human anti-protein A conjugated to horseradish peroxidase (Sigma) diluted to 1:3000 in PBS and incubated at 37°C for 1 h. The reaction was developed using *o*-phenylenediamine dihydrochloride (SigmaFAST™). Ghost membranes were prepared from blood taken from six subjects, and the ELISA was carried out as an average of three repetitions. Bovine serum albumin alone was used as a negative control to test the specificity of the CFTR antibody. Total protein was determined by the bicinchoninic acid assay (Sigma).

### Procotol 5: effect of heating on intravascular ATP *in vivo*

To gain an insight regarding the effect of heating on intravascular ATP release *in vivo*, blood samples collected from subjects undergoing graded whole-body heating were assayed immediately after collection. In brief, muscle temperature and blood samples were obtained at rest and after 6 min of moderate one-legged knee-extensor exercise (mean ± SEM, 21 ± 1 W) in the following four different thermal conditions while wearing a water-perfused suit: (i) control; (ii) mild heating; (iii) moderate heating; and (iv) severe heating. Catheters were inserted into the femoral vein of the exercising leg (left leg) and in the radial artery (right forearm) using the Seldinger technique under local anaesthesia (1% lidocaine, Hameln Pharmaceuticals, Gloucester, UK). Quadriceps muscle temperature was measured in real time (TC-2000; Sable Systems, Las Vegas, NV, USA) with a T-204A tissue-implantable thermocouple microprobe (Physitemp, Clifton, NJ, USA). Arterial and venous blood samples were collected in tubes containing stop solution (for details see section below on ‘*Blood sample treatment*’) at rest and at 5 min of exercise during each experimental condition.

### Protocol 6: effect of *in vitro* heating on heat-dependent erythrocyte ATP release from arterial and venous blood samples

The aim of this protocol was to determine any differences in ATP release from arterial and venous erythrocytes exposed to *in vitro* heating. Subjects rested in a supine position while two catheters were inserted as described in the previous subsection; one was placed into the radial artery of the right arm and the other into the median cubital vein of the left arm. Arterial and venous blood samples were collected in EDTA tubes. The RBCs were immediately isolated by centrifuging whole blood at 15,493*g* for 30 s at room temperature followed by removal of plasma. The samples were then subjected to the *in vitro* heating protocol in water baths set at 33 and 39°C for 20 min. Blood haemoglobin oxygen saturation and 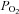 were measured immediately upon collection in separate samples using a blood gas analyser (ABL 825; Radiometer, Copenhagen, Denmark).

### Blood sample treatment

Blood samples (2 ml) obtained during the *in vivo* experiment (protocol 5) were collected directly in stop solution (2.7 ml) containing *S*-(4-nitrobenzyl)-6-thioinosine (NBTI; 5 nmol l^−1^), 3-isobutyl-1-methylxanthine (IBMX; 100 μmol l^−1^), forskolin (10 μmol l^−1^), EDTA (4.15 mmol l^−1^), NaCl (118 mmol l^−1^), KCl (5 mmol l^−1^) and tricine buffer (40 mmol l^−1^) developed by Gorman *et al.* (2003). This stop solution prevents any further release and metabolism of ATP, and values have been shown to remain stable for ∼30 min (Gorman *et al.* 2003, 2007). Blood samples were weighed to enable precise calculation of the blood volumes and determine the dilution factor for the ATP concentration. To samples undergoing the *in vitro* heating protocol, stop solution was added after 20 min of heating. Samples were immediately centrifuged for 30 s at 15,493*g*, and the supernatant was transferred into new tubes for ATP and haemoglobin determination.

### Measurement of ATP and haemoglobin

The ATP was determined with the luciferin–luciferase technique, using a luminometer with three automatic injectors (Orion Microplate Luminometer; Berthold Detection System GmbH, Pforzheim, Germany). ATP in the supernatant was measured in duplicate at room temperature (20–22°C) using an ATP kit (ATP Kit SL; BioThema AB, Dalarö, Sweden) with an internal ATP standard procedure. According to the manufacturer, free haemoglobin does not interfere with the analysis, and the minimum detectable concentration of ATP is 10^−12^ mol l^-1^, while the maximum is 10^−6^ mol l^-1^. Haemoglobin was measured to estimate the degree of haemolysis by reading the absorbance of the supernatants at 560, 577 and 593 nm (Cripps, 1968) with a spectrophotometer (Jenway 3500; Bibby Scientific, Stone, UK). Haemoglobin concentration (in grams per litre) was calculated from the absorbances (*A*) by {177.6 ×[*A*_577_– (*A*_560_+*A*_593_)/2]}, where 177.6 is the calibration coefficient *k* (Malinauskas, 1997). The effect of temperature on haematocrit was assayed by incubating whole blood for 20 min in the different water baths, at the end of which the haematocrit was determined in duplicate by microcentrifugation (Dill & Costill, 1974).

### Statistical analysis

A one-way repeated-measures ANOVA was performed on all dependent variables to test significance among the initial and heating conditions. When a significant difference (*P* < 0.05) was found, appropriate *post hoc* analysis was conducted (i.e. Tukey's HSD test) using a Bonferroni correction when the data were not normally distributed (*P* < 0.0125). Correlation between muscle temperature and plasma ATP was determined using Pearson's product–moment correlation.

## Results

### Effect of increases in temperature on ATP release from blood constituents *in vitro*

**Figure 1:**
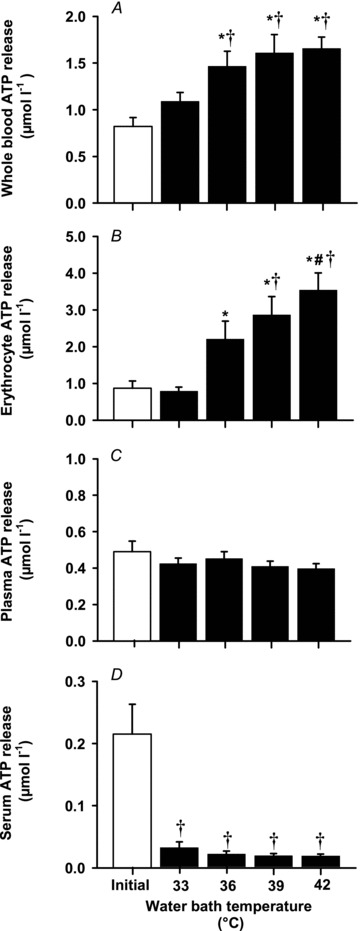
Initial ATP and the effect of increasing temperature on whole blood (*A*), erythrocytes (*B*), plasma (*C*) and serum (*D*) Open bar, initial starting ATP (*n* = 7–12); filled bars, ATP release after heating for 20 min (*n* = 7–10). Data are shown as means ± SEM. *Significantly different from 33°C (control), *P* < 0.05. # Significantly different from 36°C, *P* < 0.05. † Significantly different from initial, *P* < 0.05.

Whole blood heated for 20 min at 39 and 42°C released significantly more ATP than at 33°C, increasing from 1.10 ± 0.08 to 1.61 ± 0.20 and 1.66 ± 0.12 μmol l^−1^, respectively (*P* < 0.05; Fig. 1*A*). When compared with the initial levels, ATP release was significantly elevated at all temperatures ≥36°C. Moreover, a close correlation was observed between ATP release and water bath temperature (*r*^2^ = 0.86; *P* = 0.074).

When the RBC fraction alone was heated, the magnitude of ATP release compared with whole blood heated at the same temperatures was more than twofold higher (Fig. 1*B*). The ATP release from RBCs increased progressively and significantly from 0.79 ± 0.11 μmol l^−1^ at 33°C to 3.54 ± 0.47 μmol l^−1^ at 42°C (*r*^2^ = 0.96; *P* = 0.021). To demonstrate that the ATP was released from RBCs and not from any other blood constituents contained within the sample, RBCs were also prepared by washing three times in a buffered solution as described by Olearczyk *et al.* (2004). After heating, a similar correlation was demonstrated, where ATP release was augmented with increasing temperature from 1.18 ± 0.23 μmol l^−1^ at 33°C to 3.69 ± 0.75 μmol l^−1^ at 42°C (*r*^2^ = 0.93; *P* = 0.033; data not shown). In contrast, no changes were apparent in plasma samples, and ATP levels were significantly lower than in either whole blood or RBC samples (Fig. 1*C*).

Heat had the opposite effect on ATP release from serum, as ATP levels declined significantly in the heated serum samples compared with initial levels. Moreover, the ATP concentration in serum samples was significantly lower compared with initial levels found in whole blood, RBC or plasma (Fig. 1*D*). When considered collectively, the initial starting ATP levels were ∼1 μmol l^−1^ in whole blood and RBCs, ∼0.5 μmol l^−1^ in plasma and ∼0.2 μmol l^−1^ in serum. With heating to 42°C, ATP release nearly doubled in whole blood, increased by nearly fourfold in RBCs, remained unchanged in plasma and decreased by 10-fold in serum.

Free haemoglobin concentration remained unchanged with increasing temperature in whole blood, RBC, plasma or serum samples (*P* ranged from 0.48 to 0.98; Table 1). Likewise, whole blood haemotological responses also remained unaltered after incubation for 20 min at 33, 36, 39 and 42°C (Table 2).

**Table 1:** Free haemoglobin levels in bathing solution after heating the different components of blood

	Initial	33°C	36°C	39°C	42°C
		Free haemoglobin (g l^−1^)			
Whole blood	0.14 ± 0.01	0.20 ± 0.02	0.22 ± 0.03	0.23 ± 0.03	0.25 ± 0.03
Erythrocytes	0.18 ± 0.05	0.15 ± 0.02	0.14 ± 0.02	0.15 ± 0.02	0.17 ± 0.03
Plasma	0.07 ± 0.01	0.07 ± 0.01	0.08 ± 0.01	0.07 ± 0.01	0.07 ± 0.01
Serum	0.03 ± 0.002	0.03 ± 0.001	0.03 ± 0.002	0.03 ± 0.002	0.03 ± 0.001

Data are shown as means ± SEM of *n* = 7–10.

**Table 2:** Haematological responses after heating whole blood samples at different temperatures

	Water bath temperature (°C)				
Variables	Initial	33	36	39	42
RBCs (× 10^6^μl^−1^)	5.2 ± 0.1	5.1 ± 0.1	5.1 ± 0.1	5.1 ± 0.1	5.2 ± 0.1
Hb (g l^−1^)	155 ± 2	154 ± 1	155 ± 3	152 ± 4	156 ± 1
Haematocrit (%)	44.2 ± 0.5	44.0 ± 0.3	43.7 ± 0.9	43.4 ± 1.1	44.4 ± 0.3
MCV (fl)	85.4 ± 0.5	85.5 ± 0.2	85.8 ± 0.1	85.8 ± 0.1	85.9 ± 0.1
MCH (pg)	30.1 ± 0.1	30.0 ± 0.1	30.3 ± 0.1	30.2 ± 0.1	30.2 ± 0.1
MCHC (g l^−1^)	352 ± 1	351 ± 1	353 ± 1	352 ± 1	352 ± 2

Values are means ± SEM from one individual tested eight times. Abbreviations: RBC, red blood cells; Hb, total haemoglobin concentration; MCV, mean cell volume; MCH, mean cell haemoglobin; and MCHC, mean corpuscular haemoglobin concentration.

### Time course of the temperature-dependent release of ATP from human erythrocytes

**Figure 2:**
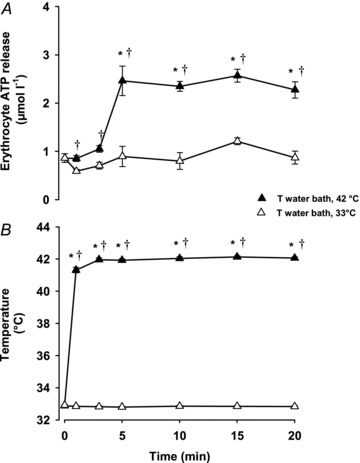
Time-dependent release of ATP from red blood cells (RBCs) Samples heated to 42°C were compared with samples maintained at a constant temperature of 33°C (*A*). The temperature of RBC samples incubated in water baths set at 33 and 42 °C (*B*). Data are shown as mean ± SEM, *n* = 6. *Significantly different from 0 min, *P* < 0.01. † Significantly higher than 33°C, *P* < 0.05.

The temperature of the RBC solution increased from 33.0 ± 0.2 to 41.3 ± 0.2 and 42.0 ± 0.1°C after 1 and 3 min and remained at ∼42°C for the remaining 15 min incubation period. In contrast, it remained at ∼33°C in the control conditions throughout the 20 min incubation period. In the heated samples, the time needed to induce a significant elevation in ATP release from RBCs was 5 min and did not increase any further for the rest of the 20 min incubation time (Fig. 2). However, when comparing differences between conditions during the first 5 min (*n* = 10), ATP release was significantly elevated at 1, 3 and 5 min at 41–42°C compared with samples maintained at 33°C (*P* ranged from 0.023 to 0.001).

**Figure 3:**
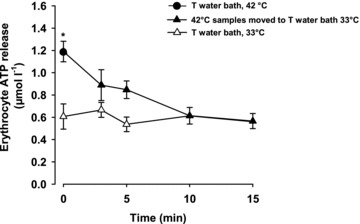
Release of ATP from RBCs with cooling after heating Samples were heated to 42°C for 20 min and then moved to 33°C. Control samples remained at 33°C throughout. Data are shown as means ± SEM, *n* = 6. *Significantly higher than 33°C, *P* < 0.05.

Conversely, when samples heated to 42°C for 20 min were moved to a water bath set at 33°C, ATP release dropped sharply, so that after 10 min it was back to similar levels of ATP found in samples that remained at 33°C (Fig. 3). These continued to remain stable after 15 min and, in further samples, even after 60 min (data not shown). Normalization of the data to the numbers of RBCs demonstrated that at 33°C the ATP release was 1.6 ± 0.2 μmol per 10^8^ RBCs, while heating to 42°C ATP release was increased to 3.4 ± 0.4 μmol per 10^8^ RBCs (*P* = 0.033). No difference in the number of red cells was observed after heating at 42 compared with 33°C (i.e. 3.7 ± 0.4 × 10^9^*versus* 3.7 ± 0.2 × 10^9^ RBCs ml^-1^, respectively; *P* = 0.84).

### Effect of ATP channel and transporter blockers on temperature-dependent release of ATP from RBCs

**Figure 4:**
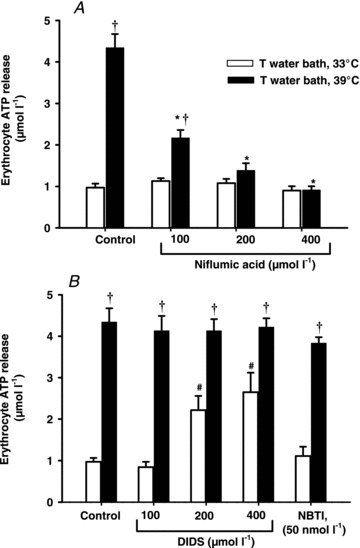
Release of ATP in the presence of niflumic acid (*A*) or DIDS or nitrobenzylthioinosine (NBTI; *B*) from erythrocyte samples heated to 39°C compared with control conditions (33°C) Open bars, ATP release at 33°C; filled bars, ATP release at 39°C. Data are shown as means ± SEM for six subjects. *Significantly lower than control 39°C, *P* < 0.05. † Significantly different from each respective condition at 33°C. # Significantly higher than control 33°C, *P* < 0.05.

An attempt to block the heat-induced ATP release from RBCs was carried out using 100, 200 and 400 μmol l^−1^ doses of niflumic acid. This was conducted at a temperature of 39°C, evoking a more than threefold increase in ATP release compared with the control conditions at 33°C (Fig. 4). Incubation of RBCs with different concentrations of niflumic acid at 33°C for 20 min did not alter the release of ATP, and values were comparable to control (Fig. 4*A*). In the presence of increasing concentrations of niflumic acid, heat-induced ATP release was significantly attenuated by 68 ± 6 to 91 ± 6% (Fig. 4*A*; all *P* < 0.01).

Use of the band 3 transporter inhibitor DIDS did not inhibit the heat-induced ATP release from RBCs even at the highest concentration of 400 μmol l^−1^ (*P* < 0.05; Fig. 4*B*), nor did DIDS increase temperature-mediated ATP release, despite its significant effect on ATP release at 33°C at the two higher concentrations, possibly due to haemolysis (Table 3). In contrast, there was a slight but not significant 18 ± 6% inhibition of ATP release in the presence of the nucleoside inhibitor NBTI. In contrast to DIDS, no significant changes in free haemoglobin were observed with niflumic acid or NBTI, at either 33 or 39°C.

**Table 3:** Free haemoglobin levels in bathing solution after heating erythrocytes treated with niflumic acid, DIDS and nitrobenzylthioinosine (NBTI)

		Haemoglobin (g l^−1^)						
		
		Niflumic acid (μmol l^−1^)			DIDS (μmol l^−1^)			NBTI (nmol l^−1^)
				
Temperature (°C)	Control	100	200	400	100	200	400	50
33	0.15 ± 0.01	0.17 ± 0.02	0.16 ± 0.02	0.13 ± 0.02	0.13 ± 0.03	0.25 ± 0.04^*^	0.32 ± 0.05^*^	0.16 ± 0.03
39	0.09 ± 0.01	0.17 ± 0.03	0.16 ± 0.02	0.13 ± 0.01	0.16 ± 0.02	0.22 ± 0.02^*^	0.32 ± 0.04^*^	0.13 ± 0.02

Data are shown as means ± SEM, of *n* = 6. ^*^Significantly different from control value *P* < 0.05.

### Effect of CFTR inhibition with glibenclamide on temperature-dependent release of ATP from RBCs

**Figure 5:**
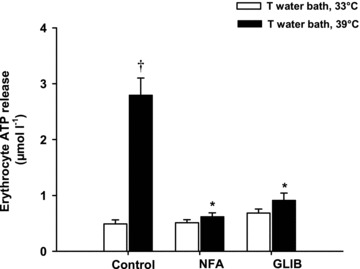
Release of ATP by venous erythrocyte samples heated to 39°C compared with control conditions (33°C) in the presence of 400 μmol l^−1^ niflumic acid (NFA) or 400 μmol l^−1^ glibenclamide (GLIB) Open bars, ATP release at 33°C; filled bars, ATP release at 39°C. Data are shown as means + SEM for nine subjects. * Significantly lower than control 39°C, *P* < 0.05. † Significantly different from control conditions at 33°C.

To assess the involvement of CFTR on temperature-sensitive ATP release, RBCs were incubated in the presence of glibenclamide. In the same samples of RBCs, the effect of 400 μmol l^−1^ niflumic acid was also demonstrated. Both glibenclamide and niflumic acid completely blocked the release of ATP (*P* < 0.05; Fig. 5). Similar to niflumic acid, a dose-dependent inhibition with was observed glibenclamide, with a reduction starting at 100, 200 and 400 μmol l^−1^ (data not shown). Free haemoglobin levels in the presence of either niflumic acid or glibenclamide were unchanged at both 33 and 39°C (∼0.1 ± 0.01 g l^−1^).

### Abundance of CFTR protein in RBCs subjected to heating

**Figure 6:**
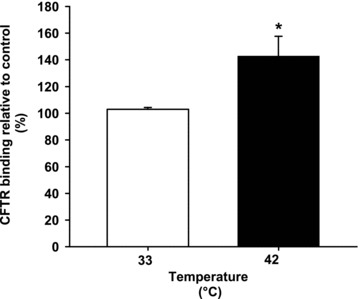
ELISA analysis of the binding capacity of CFTR on protein isolated from RBC ghost membranes heated at 42°C relative to 33°C (control) Open bars, CFTR binding at 33°C; filled bars, CFTR binding at 42°C. Data are shown as means ± SEM for six subjects. * Significantly different from 33°C (control).

To confirm the involvement of CFTR in temperature-dependent ATP release, protein abundance in ghost membranes was assessed by ELISA. In RBCs heated at 33 and 42°C isolated from blood from six individuals, CFTR abundance increased by 42 ± 14% (*P* < 0.05; Fig. 6). Protein determined by bicinchoninic acid assay confirmed that the concentrations of total protein in ghost membrane samples incubated at 33 and 42°C were similar (5.0 ± 0.5 and 4.9 ± 0.5 mg ml^−1^, respectively). Further evidence of the effect of temperature on the abundance of CFTR in RBC membranes was observed when samples were cooled down to 33°C for 15 min after heating at 42°C for 20 min. The CFTR abundance in these samples prepared from blood taken in two individuals declined towards control values.

### Effect of increases in temperature on arterial and venous plasma ATP *in vivo*

**Figure 7:**
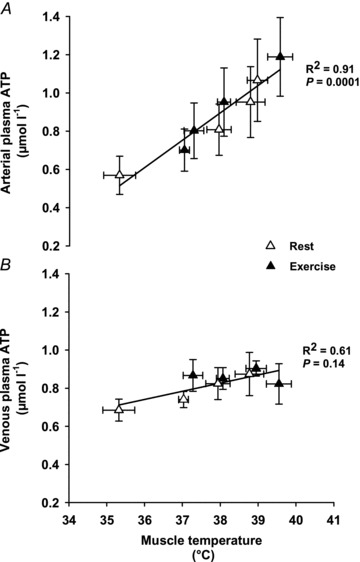
Relationship between quadriceps muscle temperature and plasma ATP Whole-body heat stress increases arterial plasma ATP levels with both rest and exercise (*r*^2^= 0.91; *P* < 0.0001). No significant relationship was observed between venous plasma ATP levels and muscle temperature (*r*^2^= 0.61; *P*= 0.14). Open triangles represent rest, whereas closed triangles represent exercise measurements. Data are means ± SEM for 10 subjects.

Graded whole-body heating in human participants increased quadriceps muscle temperature at rest from 35.3 ± 0.4°C in control conditions to 37.0 ± 0.1°C with mild heating, 37.9 ± 0.3°C with moderate heating and finally to 38.8 ± 0.4°C with severe heating. During exercise, the equivalent values were 37.3 ± 0.3°C in control conditions 38.1 ± 0.2°C with mild heating, 38.9 ± 0.3°C with moderate heating and 39.6 ± 0.3°C with severe heating. A significant correlation was observed between the rise in muscle temperature and the increase in arterial plasma ATP during both rest and exercise (*r*^2^ = 0.91; *P* = 0.0001; Fig. 7). This relationship was attenuated in venous plasma ATP samples taken during rest and exercise (*r*^2^ = 0.61; *P* = 0.137). Arterial or venous plasma haemoglobin remained unchanged with graded whole-body heating, indicating that haemolysis did not contribute to the increase in plasma ATP (∼0.1 ± 0.01 g l^−1^ for both arterial and venous samples).

### Effect of increases in temperature on ATP release from erythrocytes in arterial and venous samples *in vitro*

**Figure 8:**
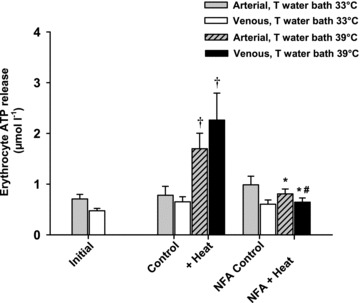
Release of ATP from arterial and venous erythrocyte samples heated to 39°C compared with control conditions (33°C) in the presence of 400 μmol l^−1^ NFA Grey bars, arterial ATP release at 33°C; open bars, venous ATP release at 33°C; grey hatched bars, arterial ATP release at 39°C; filled bars, venous ATP release at 39°C. Data are shown as means ± SEM for four subjects. Significantly different from venous initial. † Significantly higher than venous initial, venous control at 33°C, venous NFA at 33°C and venous NFA at 39°C. # Significantly lower than arterial control at 39°C.

The ATP release was compared in RBCs heated *in vitro* at 33 or 39°C from arterial and venous blood samples taken in control resting conditions. The haemoglobin oxygen saturation in the arterial and venous blood was 98.5 ± 0.3 and 51.2 ± 6.2%, respectively, with corresponding 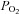 values of 105 ± 1 and 35 ± 4 mmHg. The release of ATP was comparably increased to 1.69 ± 0.30 and 2.26 ± 0.53 μmol l^−1^ (*P* < 0.05) in both arterial and venous samples heated at 39°C from 0.78 ± 0.17 and 0.65 ± 0.09 μmol l^−1^ at 33°C, respectively (Fig. 8). Furthermore, the responses of both arterial and venous RBCs to niflumic acid were similar, indicating that the sensitivities of the temperature-sensitive ATP transporters were not affected by the oxygenation status of the RBCs, because they demonstrated complete inhibition (*P* < 0.05; Fig. 8).

## Discussion

This study reveals six key findings that provide novel insight into the blood source and the temperature-sensitive mechanisms of ATP release in human red blood cells. First, the erythrocytes were the primary source of ATP from blood-formed elements. Second, cooling after heating restored erythrocyte ATP release to baseline levels. Third, heat-mediated erythrocyte ATP release was completely blocked by either niflumic acid (a non-specific ion channel inhibitor which blocks Ca^2+^-activated Cl^−^ channels) or glibenclamide (an inhibitor of CFTR) but was not blocked by inhibitors of nucleoside transport or anion exchange. Forth, heating the blood enhanced protein abundance of CFTR in membrane protein fractions isolated from RBCs. Fifth, *in vitro* findings were consistent with the progressive increase in arterial plasma ATP observed *in vivo* in resting and exercising heat-stressed humans. Sixth, *in vitro*, however, the increase in ATP release from RBCs was similar in arterial and venous samples, suggesting that the lower *in vivo* rate of increase in venous plasma ATP with temperature might be due to increased ATP degradation. These findings collectively demonstrate that erythrocyte ATP release is sensitive to physiological increases in temperature via CFTR-like channels and imply the existence of a potentially important mechanism for control of local tissue perfusion in conditions of elevated blood and tissue temperature.

### Source of temperature-dependent ATP release in human blood

A key aim of the present study was to identify the source of ATP release from blood components. With this aim, we used an *in vitro* experimental set-up that excluded the potential confounding influences of endothelial and sympathetic nerve-derived ATP. The temperatures used to heat whole blood, RBCs, plasma and serum (33–42°C; Fig. 1) cover the normal physiological temperature range found in the human blood and muscle tissue during exposure to environmental heat stress, external heating and/or exercise (Saltin *et al.* 1972; González-Alonso *et al.* 1999*a*,*b*). In the present *in vivo* study specifically, quadriceps muscle temperature ranged from 34°C in the resting control conditions to 41°C during combined heat stress and knee-extensor exercise (Fig. 7). Thus, the design of this study also allowed the comparison of the effect of graded increases in temperature on ATP release from whole blood and its individual constituents *in vitro* to the effect of whole-body heating *in vivo*.

An important observation of this study was that ATP release from heated whole blood *in vitro* was significantly elevated and, interestingly, reached comparable levels to those found in the arterial plasma *in vivo* even though the experimental conditions were vastly different. A critical question is, which blood constituent accounts for the increase in ATP? To answer this question, heat-induced ATP release was measured from RBCs, plasma and serum samples. We observed that ATP release from RBCs increased progressively with temperature (*r*^2^ = 0.96; *P* = 0.021) to levels higher than in whole blood or in plasma during *in vivo* heating and exercise. In contrast, heating plasma or serum did not elevate the free ATP concentration with increasing temperature from 33 to 42°C (Fig. 1*C*.). However, and in contrast to whole blood, RBCs and plasma, a significant fall in serum ATP was observed from the initial basal concentration of 0.215 μmol l^−1^ to >0.019 μmol l^−1^ at all temperatures. This indicates the presence of a high level of ATP catabolic activity within the serum fraction in the form of the soluble enzymes nucleoside triphosphate diphosphohydrolase (NTPDase; known as ecto-ATPDase, CD39), nucleotide pyrophosphatase (NPP) and hydrolase plus ecto-5′-nucleotidase (Yegutkin *et al.* 2003), which all work together to break down ATP. The difference in catalytic activity of plasma ecto-5′-nucleotidase might account for the drop in ATP levels in serum and the maintenance of ATP in cell-free plasma (Coade & Pearson, 1989).

Critical to the interpretation of the present findings is whether the observed increase in plasma or ‘bathing solution’ ATP with elevations in temperature is due to haemolysis and thus contamination from the higher intracellular ATP concentration in RBCs. To avoid this possibility, blood samples were handled carefully while centrifuging and pipetting, and the isolation procedure did not include a wash step or resuspension step in a buffer solution. In our study, plasma or ‘bathing solution’ free haemoglobin concentration remained unchanged with increases in temperature from 33 to 42°C in RBC preparations (Table 1) and thus the temperature-dependent increase in ATP was not correlated to free haemoglobin (*r*^2^ = 0.48; *P* = 0.921). Furthermore, RBC count, mean cell volume, mean cell haemoglobin and mean corpuscular haemoglobin concentration did not change, further suggesting that the rise in temperature did not affect the volume and integrity of the blood cells. In addition, we reason that if the ATP release was due solely to haemolysis, rather than a transport-mediated process, it would not respond to any blocking agent. In support of a transport-mediated process, we found that ATP release was completely blocked with 400 μmol l^−1^ niflumic acid or 400 μmol l^−1^ glibenclamide at 39°C (Figs 4*A*, 5 and 8). Therefore, the short time for which the erythrocytes were exposed to temperatures of >36°C in the present study did not cause any statistically significant haemolysis. Moreover, any remaining plasma and serum in the RBC fraction would contribute very little to the observed fourfold increase in heat-induced release of ATP from RBCs (Fig. 1*C* and *D*). Taken together, these findings demonstrate that the sole blood source of temperature-dependent ATP release is the erythrocytes, and the contribution from other elements within the blood is negligible.

Another key question is, how rapidly does temperature increase ATP release from erythrocytes? As a first attempt to answer this question, we heated the RBC solutions to ∼42°C for different durations (1, 3, 5, 10 and 20 min) and compared results with control conditions at ∼33°C. In this experimental setting, we observed a significantly higher [ATP] in the ‘bathing solution’ after 1 min of incubation at ∼42 compared with ∼33°C and a significant increase in ATP over time after 5 min in the heated conditions. Although studies measuring ATP release in real time with sensors specific for ATP are required to assess the temporal response to heating more precisely (Llaudet *et al.* 2005), we believe that the observed differences in ATP after 1 min of heating might have important regulatory implications *in vivo*. For instance, in our recent isolated leg heating study (Pearson *et al.* 2011) the leg blood flow increased progressively from 0.5 ± 0.1 to 1.0 ± 0.1 l min^-1^ over the 1 h heating protocol, which was closely associated with the gradual increase in muscle temperature. Moreover, we have previously shown that a very low ATP infusion rate of 200 nmol min^-1^ into the femoral artery, which does not change plasma [ATP], can increase leg blood flow by 1 l min^-1^ (González-Alonso *et al.* 2002). It is therefore reasonable to suggest that increases in intravascular ATP have the potential to contribute to the vasodilatation in the vasculature of the leg tissue seen during local heating *in vivo*. Another important observation of this study was the effect of cooling after heating, showing that ATP release gradually returned to control values (Fig. 3). This further supports the suggestion that heating *per se* does not disturb the integrity of the RBCs or the functioning of the ATP channels, and thus erythrocyte ATP release is a true physiological response triggered by heating.

### Mechanisms of temperature-dependent ATP release from human erythrocytes

The present findings demonstrate that temperature is an important physiological stimulus for the release of ATP from human erythrocytes. A second aim of this study was to gain insight into the temperature-sensitive mechanisms of ATP release. There are several possible pathways by which temperature might operate. First, increases in temperature could affect the fluidity of the erythrocyte membrane, which in turn could alter the lipid and protein interactions and increase permeability for ATP and other molecules. Although this is a reasonable possibility when exposing RBCs to very high temperatures (i.e. >48°C), its relevance at the temperatures used in this study (33–42°C) seems to be minimal (Williamson *et al.* 1975), because temperature-mediated erythrocyte ATP release was completely blocked with either niflumic acid or glibenclamide, despite the persistent high temperature, and was fully restored with application of cooling after heating. Second, ATP could be released by diffusion down a gradient, because the intracellular concentration of ATP in RBCs is ∼2 mmol l^−1^, whereas the plasma concentration in resting humans is normally in the nanomolar range (González-Alonso *et al.* 2002; Rosenmeier *et al.* 2004; Yegutkin *et al.* 2007; Dufour *et al.* 2010; Mortensen *et al.* 2011). However, this is also an unlikely possibility, because ATP is a large, charged molecule and thus would not easily cross the cellular membrane (Gordon, 1986). A third, more likely possibility is that an active transporter or channel is responsible for the controlled, temperature-dependent movement of ATP out of the erythrocyte.

Release of ATP from erythrocytes has been proposed to involve membrane-bound ion channels, gap junction proteins, such as pannexin 1, and/or the ATP-binding cassette proteins, such as CFTR (Bergfeld & Forrester, 1992; Abraham *et al.* 1993; Locovei *et al.* 2006). There are three observations in the present study that provide strong support for the involvement of CFTR or CFTR-like channels. First, we found that the temperature-mediated erythrocyte ATP release was completely blocked with the inhibitors of Ca^2+^-activated Cl^−^ channels niflumic acid and glibenclamide (a CFTR inhibitor), but not with DIDS. This suggests the involvement of a Cl^−^ channel that is insensitive to DIDS, contrary to the study by Bergfeld & Forrester (1992). The solubility of the different blockers could be increased by temperature, and other unspecific properties, such as lysis caused by DIDS, could be resolved by washing away and then measuring the effects. However, the temperature-sensitive channel proposed in the present study could be similar to the erythrocyte channel stimulated by deformation, also thought to be CFTR, because both niflumic acid and glibenclamide inhibited ATP release (Sprague *et al.* 1998). Furthermore, previous work has demonstrated that niflumic acid can also directly block the CFTR channel by inhibiting the permeation of Cl^-^ currents by plugging the channel pore (Scott-Ward *et al.* 2004). Second, the presence of NBTI did not block the heat-mediated ATP release from the erythrocytes. This provides further evidence that the temperature-sensitive channels in erythrocytes are not affected by inhibitors of either nucleoside transport or anion exchange (Bergfeld & Forrester, 1992). Finally, CFTR protein abundance was increased, possibly due to temperature-induced trafficking to the cell membrane or demasking of epitope regions, because erythrocytes do not have the necessary tools for protein synthesis (Denning *et al.* 1992; Cheng *et al.* 2010). This temperature-mediated CFTR translocation or activation could facilitate the transport of ATP out of the RBCs. This is consistent with the observation that ATP release induced by decreasing pH also increased CFTR abundance in isolated skeletal muscle cells (Tu *et al.* 2010). Taken as a whole, these observations indicate that the increase in temperature-stimulated erythrocyte ATP release might involve the activation of erythrocyte CFTR or CFTR-like channels.

An alternative physiological mechanism that could play a part in promoting the release of ATP when temperature is increased is the shift in the oxyhaemoglobin dissociation curve (Barcroft & King, 1909; Duc & Engel, 1969). A rightward shift of the curve reflects a reduction in the affinity of haemoglobin for oxygen that could lead to an augmented oxygenation-coupled ATP release from erythrocytes. Pioneering work demonstrated that erythrocytes release ATP in response to hypoxia or independent reductions in 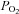 and haemoglobin oxygenation (Bergfeld & Forrester, 1992; Ellsworth *et al.* 1995; Sridharan *et al.* 2010). An important question is therefore whether the presently observed increases in erythrocyte ATP release with progressive elevations in temperature are influenced by the oxygenation state of the haemoglobin molecule and parallel changes in 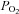. The evidence from our study suggests that the temperature stimulus for erythrocyte ATP release differs from the well-characterized stimulus coupled to the fall in 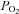 and oxyhaemoglobin (Ellsworth *et al.* 1995). For instance, the parallel observation that femoral venous oxygenation and 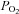 increased during whole-body heating *in vivo* accompanying an elevation of leg tissue blood flow, but arterial oxygenation remained unchanged and arterial ATP increased gradually with the rise in muscle temperature (Pearson *et al.* 2011), supports a pathway of ATP release that is independent of reductions in blood oxygenation. This notion is further supported by the present *in vitro* observation showing similar initial and control ATP levels in arterial and venous plasma despite large differences in haemoglobin oxygen saturation and 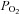 (i.e. 98 *versus* 55% and 105 ± 1 *versus* 35 ± 4 mmHg). Furthermore, any differences with regard to affinity of arterial or venous blood to the presence of niflumic acid were not apparent, because they were both equally blocked to prevent heat-induced ATP release. Taken together, the present findings highlight a novel mechanism of temperature-dependent ATP release from erythrocytes, which is independent of the oxygen offloading from the haemoglobin molecules.

### Physiological implications for the control of local tissue perfusion

This study provides an insight into a potential mechanism involved in the local control of blood flow during conditions of increasing blood and tissue temperature, such as exposure to heat stress, exercise, fever or thermal therapy. Although increases in limb blood flow with heat stress have generally been thought to be the sole response to an augmented thermoregulatory demand of the skin circulation (Rowell, 1974), evidence in pigs and humans shows that skeletal muscle blood flow is also increased with local and whole-body heating (Akyurekli *et al.* 1997; Keller *et al.* 2010; Pearson *et al.* 2011), a response that is closely related to the rise in local muscle temperature and the arterial plasma concentration of ATP during heat stress at rest and during exercise (Pearson *et al.* 2011). Knowing that muscle and capillary blood temperatures are similar due to rapid temperature equilibration (He *et al.* 2002), our parallel and present observations indicate that local hyperthermia induces vasodilatation through increases in intravascular ATP and/or other vascular signals sensitive to temperature. ATP is an attractive mediator signal for skeletal muscle blood flow control, because not only can it act as a potent vasodilator, but also it has sympatholytic properties in the leg and forearm (Rosenmeier *et al.* 2004; Kirby *et al.* 2008), which are required to maintain or increase perfusion in conditions of augmented muscle sympathetic nerve activity, such as heat stress and exercise (Niimi *et al.* 1997; Ray & Gracey, 1997; Pearson *et al.* 2011).

The temperature-dependent mechanism of ATP release from human erythrocytes documented in this study opens the possibility of using external heating as a non-pharmacological means of increasing limb tissue perfusion, including that to skeletal muscle, in diseases characterized by limb ischaemia. It is known that circulatory problems associated with ageing and a number of diseases, such as diabetes and peripheral vascular disease, could benefit from increasing flow to limb tissues that become deprived of oxygen and substrates (Feliciano & Henning, 1999; Schrage *et al.* 2007; Black *et al.* 2008; Kirby *et al.* 2010; Ruiter *et al.* 2010). Although decreased cell-to-cell communication along vascular endothelium, changes in receptor expression or damaged endothelium, and impaired smooth muscle function could potentially blunt the limb tissue hyperaemic response to heating in some patient populations, studies using thermal therapy or ‘forearm heat training’ have shown profound muscle hyperaemia and beneficial endothelial adaptive responses (Akyurekli *et al.* 1997; Green *et al.* 2010). It would therefore be interesting to extend these studies by investigating whether limb heating enhances ATP release from erythrocytes and increases skeletal muscle blood flow in elderly people and different patient populations with circulatory dysfunction.

In summary, our *in vitro* and *in vivo* findings demonstrate that erythrocyte ATP release is sensitive to physiological increases in temperature independent of oxygenation, possibly by activation of a CFTR-like channel. These data have potential clinical implications for the treatment of patients with peripheral vascular disease non-pharmacologically, by using local heating to stimulate erythrocyte ATP release and thus increase flow and oxygen and substrate supply to limb tissues.
